# Protocol for a phase II interventional trial investigating indocyanine green (ICG) fluorescence guided lymph node mapping for determination of bowel resection margins in colon cancer (ISCAPE)

**DOI:** 10.1097/SP9.0000000000000041

**Published:** 2025-03-20

**Authors:** Lidiia Panaiotti, Aleksei Karachun, Anastasia Muravtseva, Aleksei Petrov

**Affiliations:** a Surgical Emergency Unit, John Radcliffe Hospital, OUH, Oxford, United Kingdom; b Surgical Department of Abdominal Oncology, FSBI “N.N. Petrov National Medical Research Center of Oncology” of the Ministry of Healthcare of Russian Federation, Saint Petersburg, Russian Federation; c Department of Pathology, FSBI “N.N. Petrov National Medical Research Center of Oncology” of the Ministry of Healthcare of Russian Federation, Saint Petersburg, Russian Federation; d Department of Surgery, Royal Bournemouth Hospital, University Hospitals Dorset, Bournemouth, United Kingdom

**Keywords:** colon cancer, complete mesocolic excision (CME), fluorescence imaging, fluorescence lymphangiography, indocyanine green (ICG), lymphatic mapping

## Abstract

**Background:**

Optimal extent of lymph node dissection for colon cancer is debatable. Extensive lymphadenectomy may increase complication rate, while limited lymph node dissection may compromise oncological outcome. One of promising ways to find balance is to tailor extent of lymph node dissection to patient’s individual anatomy using ICG lymphatic mapping.

**Methods:**

This is a single center interventional phase II trial with single group assignment aiming to determine if ICG lymphatic mapping sensitivity is sufficient to guide resection margins selection in colon cancer surgery. The trial’s primary endpoint is proportion of pN+ patients in which affected lymph nodes are detected only within margins of ICG spread. Sample size of 101 patients was calculated using Buderer method ^[19]^ with a confidence level (1 − *α*) of 0.95 as a minimum of cases required to test accuracy of lCG lymphatic mapping for estimated sensitivity of 0.99 and precision of 0.03. The average of pN+ cases in our center (42%) was used as prevalence. Secondary endpoints are incidence of adverse events related to ICG lymphatic mapping, feasibility of ICG lymphatic mapping for colon cancer, incidence of lymph node metastases outside conventional resection margins (10 cm), colon cancer lymphatic spread patterns, proportion of operations which extent is affected by ICG lymphatic mapping. The trial is conducted among female or male patients, 18 years or older, with signed informed consent, and diagnosed primary colon cancer. Inclusion criteria include pathologically confirmed adenocarcinoma of the colon, T1-4aN0-2bM0-1b, clinical indications to colonic resection, ECOG – 0–2. Exclusion criteria consist of acute bowel obstruction, bleeding or perforation, adjacent organ invasion or peritoneal carcinomatosis, and contraindications to ICG administration. Eligible patients are allocated for colonic resection with intraoperative ICG mapping. During pathological examination, lymph nodes are assessed for presence of metastases and location in relation to tumor and fluorescence margins. The study began on 26 July 2022 and is conducted in and financed by N.N. Petrov NMRC of Oncology in Saint Petersburg, Russia, it is conducted in.

**Results:**

If after 101 ICG lymphatic mapping procedures, sensitivity of >96% is observed, this will provide rationale behind tailoring resection margins to fit ICG spread.

**Conclusions:**

ICG lymphangiography allows a surgeon to see locoregional lymphatics of a tumor site in real time and tailor colon and mesentery resection margins to meet oncological and functional needs. More data is needed to make this approach more widespread.

## Introduction

### Background and rational

Navigational surgery including mapping and tailoring of resection margins can be a solution to the major problem of colon cancer surgery: finding “the least possible not to compromise oncological safety” resection clearance for each patient. Mentioned concept has already been discussed^[1,2]^. But although the idea that all patients are different and therefore require different approaches sounds reasonable, this notion has not met sufficient response because yet there is no tool with proven effectiveness to implement it.

However, there is a lot of evidence on ICG fluorescent imaging for visualization of locoregional lymphatic paths of colonic lesions^[2,3]^, which can guide tailoring. ICG was firstly implemented as a sentinel lymph node detection (SLND) tool but was found to lack accuracy and standardization. Ankersmit *et al* analyzed 8 studies describing 227 SLN procedures in a meta-analysis in 2019 and observed low pooled sensitivity of 63%, negative predictive value 81% and detection rate of 94%^[4]^. The same year Villegas-Tovar *et al* reported similar findings in another meta-analysis of ICG SLND accuracy for colon cancer: they calculated detection rate of 91% (80–98%), sensitivity of 64.3% (51–76%), and specificity of 65% (36–85%) – and concluded that overall ICG performance for detection of lymph node metastases is poor^[5]^. So, it was shown that ICG does help to visualize lymphatics, but it does not work for SLND in colon cancer.

Moreover, assessment of fluorescence and fluorescent lymph node harvest are affected by multiple factors. In particular, 2023 systematic review and meta-analysis of 58 prospective designed studies by Lucas *et al* showed that injection time had a significant impact on aberrant drainage detection favoring preoperative tracer injection^[6]^. The same year Kinoshita *et al* in their series of 56 cases observed both cases with metastatic lymph nodes only in the area along the ICG-stained vascular pedicles and lymph node metastasis in areas along the ICG-unstained vascular pedicles^[7]^.

This situation can be explained by the fact that presence of fluorescence is affected by tumor factors. Kakizoe *et al* compared distribution of cancer sites in lymphatic nodes according to hematoxylin and eosin staining to fluorescent picture: lymph nodes occupied by cancer cells up to 90–100% showed no fluorescence, and in nodes with smaller proportion of occupation fluorescence was missing in cancer sites^[8]^. The same pattern was observed by Ushijima *et al*, who also reported negative correlation between such factors as tumor’s depth of invasion and presence of fluorescence, and pN+ and presence of fluorescence respectively^[9]^. So, in cases of colon cancer focusing on fluorescence of specific lymphatic nodes, or seeking for sentinel nodes with ICG lymphangiography does not seem to be a reliable approach.

On the contrary, ICG imaging of the whole locoregional lymphatic collector is beneficial. Repeatedly reported data on surgical results after ICG mapping suggest that lymph node mapping without focusing on specific nodes might be useful for identifying resections’ extent during operations for colon cancer. In a series of 18 patients, Cahill *et al* observed 22% of fluorescing lymphatic nodes outside the initial resection plan^[10]^. Nishigori *et al* reported ICG lymph node mapping led to 23.5% change in the extent of lymphadenectomy, and 16.7% change in the colon resection plan^[11]^. Moreover, Watanabe *et al* reported that in cases of central vascular ligation, the use of ICG helped to identify appropriate central vessels to be dissected, as well as to determine the appropriate separation line of mesentery so that the whole observed lymph flow is included into specimen^[12,13]^. And Daibo *et al* showed that indocyanine green fluorescence imaging-guided laparoscopic right-sided colectomy could increase the number of total, intermediate, and central lymph nodes retrieved^[14]^. Thus, it seems that multiple groups of patients are likely to benefit from ICG lymphatic mapping, because this technique provides a possibility to tailor resection margins so that they fulfill oncological demand of regional lymphatic flow removal. Trial registration information is presented in Table 1.

**Table 1:** Trial registration

Primary registry and trial identifying number	ClinicalTrials.gov NCT05468827
Date of registration in primary registry	21.07.2022
Source of material support	FSBI "NMRC of Oncology named after N.N. Petrov" of the MoH of the Russian Federation
Primary sponsor	FSBI "NMRC of Oncology named after N.N. Petrov" of the MoH of the Russian Federation
Contact for public queries	Aleksei Karachun dr.a.karachun@gmail.com
Contact for scientific queries	Aleksei Karachun dr.a.karachun@gmail.com
Title	ICG Fluorescence Guided Lymph Node Mapping for Determination of Bowel Resection Margins in Colon Cancer (ISCAPE)
Countries of recruitment	Russian Federation
Problem studied	Surgical treatment for colon cancer
Intervention	Lymphatic mapping by ICG
Key eligibility criteria	Adult female or male, 18 years or older, with signed informed consent, and diagnosed primary colon cancer.
	Inclusion Criteria: pathologically confirmed adenocarcinoma of the colon, T1-4aN0-2bM0-1b, clinical indications to colonic resection, ECOG – 0–2.
	Exclusion Criteria: acute bowel obstruction, bleeding or perforation, adjacent organ invasion or peritoneal carcinomatosis, contraindications to ICG administration.
Study type	Interventional with single group assignment
	Allocation: N/A
	Primary purpose: diagnostic
	Phase II
Date of first enrolment	26 July 2022
Target sample size	101 patients
Recruitment status	Recruiting
Primary endpoint	Proportion of pN+ patients with affected lymph nodes detected only within margins of ICG distribution
Secondary endpoints	Incidence of adverse events related to ICG lymphatic mapping, feasibility of ICG lymphatic mapping for colon cancer, incidence of lymph node metastases outside conventional resection margins (10 cm), colon cancer lymphatic spread patterns, proportion of operations which extent is affected by ICG lymphatic mapping
Protocol version	v. 3.1

### Objectives and study hypothesis

**Figure 1.**
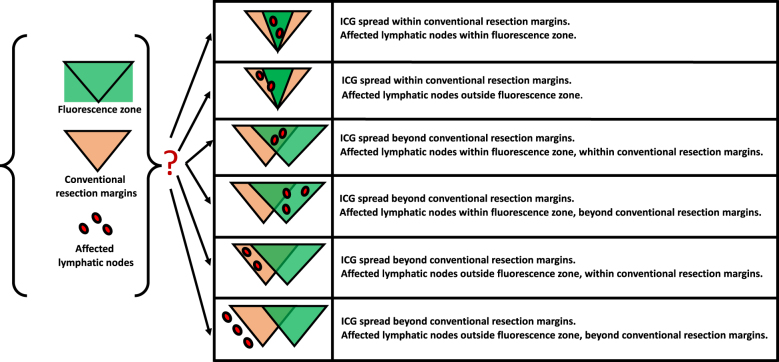
Possible relations between ICG lymphatic spread, conventional resection margins and lymphatic node metastatic involvement.

In this trial it is investigated whether the zone of lymphatics’ fluorescence visualized after subserosal ICG injection reflects the region of metastatic spread from a colon segment affected by a tumor. Even though it is assumed that ICG fills in the whole regional lymphatic collector (with all affected nodes), hypothetically, ICG mapping procedure might lead to one of six scenarios (Fig. 1): ICG spreads within conventional resection margins (affected lymph nodes within/outside fluorescence zone), or ICG spreads beyond conventional resection margins (affected lymph nodes within fluorescence zone and within/beyond conventional resection margins), or ICG spreads beyond conventional resection margins (affected lymph nodes outside fluorescence zone and within/beyond conventional resection margins). So, these groups’ detection and analysis will help to make important assumptions and considerations regarding role of ICG lymphatic mapping in colon cancer surgery. For instance, if no affected lymph nodes are observed beyond margins of ICG spread in subcategory of patients with fluorescent zone smaller than conventional resection margins, it will mean that extent of colon and mesentery resection plan can be decreased to fit ICG spread. And on the contrary, if frequency of lymph node metastases beyond ICG spread margins makes method’s sensitivity suboptimal, efficacy of ICG lymphatic mapping will have to be reconsidered.

### Aims of the study

#### Primary aim of the study

To determine if ICG lymphatic mapping sensitivity is sufficient to guide resection margins selection in colon cancer surgery.

#### Secondary aims of the study

To determine safety and feasibility of ICG lymphatic mapping, incidence of lymph node metastases outside conventional resection margins (10 cm), colon cancer lymphatic spread patterns and proportion of operations which extent is affected by ICG lymphatic mapping.

### Trial design

#### Design

This is an interventional phase II trial with single group assignment investigating feasibility of defining colon resection margins for colon cancer with ICG by comparing lymphatic distribution of subserosally injected dye with actual spread of lymphatic metastases reported by pathologists after specimen examination.

This is a single center trial. To ensure adherence to the protocol and to avoid logistical and monitoring issues stemming from multicenter design, all patients are enrolled in one center. The study includes ICG lymphatic mapping procedure and extensive pathological evaluation of the specimen, which are not routinely performed in majority of centers. Also, the calculated sample size of 101 is a number of patients, which is possible to enroll in a single center during reasonable time period. So, the risks of type II error, methodological error leading to bias and contradicting results issue, resulting from single center approach were acknowledged and study protocol created to possibly minimize them and ensure that the statistical hypothesis is tested.

Since the main focus of the study is investigation of ICG lymphatic mapping feasibility and currently there is no gold standard of lymphatic mapping for colon, no control group was included.

**Figure 2.**
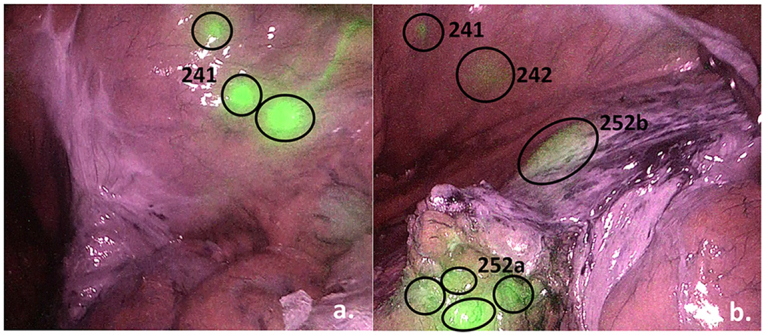
Example of fluorescence of lymph node groups recorded during surgical procedure (sigmoid resection case). Fluorescence of paracolic nodes (a.) and along vascular tie (b.) is recorded and depicted to ensure proper lymphatic node group and ICG spread marking after specimen removal.

**Figure 3.**
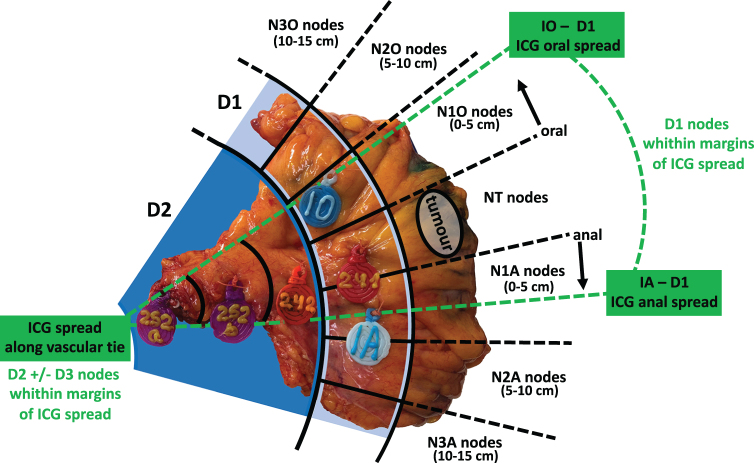
Example of specimen marking (sigmoid resection). Tags are attached to the specimen according to Japanese classification of lymphatic node groups^[15]^. Segments of D1 collector are named according to Japanese Classification of pericolic lymphatic collector groups^[16]^. IO and IA marks are borders of D1 ICG spread visualized during surgical procedure. So, the area of ICG fluorescence is projected on lymphatic node groups harvested during surgery.

After signing an informed consent and undergoing screening procedure eligible patients are assigned for colonic resection with ICG lymphatic mapping. The fluorescence is assessed before specimen removal (Fig. 2) and its mesenteric distribution is marked by surgeon on the specimen (Fig. 3). During pathological examination each lymph node is assessed both for presence of metastases and for its exact location in relation to margins of ICG distribution (each lymphatic node is classified according to Japanese classification of lymphatic node groups^[15]^; D1 nodes are also classified according to their distance from primary tumor^[16]^). All screening, intraoperative, postoperative, and pathological data is recorded for further analysis. Protocol synopsis is presented in Table 2.

**Table 2:** Protocol synopsis

Title	ICG fluorescence guided lymph node mapping for determination of bowel resection margins in colon cancer
Study design	Patients with primary resectable colon cancer are assigned for colonic resection with intraoperative ICG lymphatic mapping. The dye’s mesenteric distribution is marked by surgeon on the specimen. During pathological examination each lymph node is assessed both for presence of metastases and for its exact location in relation to primary tumor and margins of indocyanine distribution.
Aims and endpoints	*Aim of the study:*
	To determine if ICG lymphatic mapping sensitivity is sufficient to guide resection margins selection in colon cancer surgery by comparing lymphatic distribution of subserosally injected dye to the actual spread of lymphatic metastases reported by pathologists after specimen examination.
	*Primary endpoint:*
	Proportion of pN+ patients with affected lymph nodes detected only within margins of ICG distribution.
	*Secondary endpoints:*
	incidence of adverse events related to ICG lymphatic mapping; feasibility of ICG lymphatic mapping for colon cancer (proportion of ICG spillage affecting interpretation); incidence of lymph node metastases outside conventional resection margins (10 cm); colon cancer lymphatic spread patterns; proportion of operations which extent is effected by ICG lymphatic mapping.
Patients	Female and male patients 18 years or older with signed informed consent and diagnosed primary colon cancer T1-4aN0-2bM0-1b
Number of patients	101
Study duration	The study is conducted starting from 26 July 2022, until target number of patients is enrolled
Estimated time until target number of patients is reached	2 years
Statistical analysis	If after 101 ICG lymphatic mapping procedures we record sensitivity of 96% or higher, it will mean that this method might be considered as a reliable tool to visualize locoregional lymphatics of a colon segment affected by a tumor.

There is no blinding of investigators or patients in current study.

#### Endpoints

Primary endpoint

- Proportion of pN+ patients in which affected lymph nodes are detected only within margins of ICG distribution (number of patients with affected lymph nodes located within ICG distribution margins divided by the number of all patients with pN+).

Secondary endpoints

- Incidence of adverse events related to ICG lymphatic mapping (time frame: 30 days after operation);
- feasibility of ICG lymphatic mapping for colon cancer (proportion of cases with ICG spillage affecting interpretation);
- incidence of lymph node metastases outside conventional resection margins – 10 cm (proportion of patients with aberrant lymphatics leading to affected lymph nodes);
- colon cancer lymphatic spread pattern (descriptive data on incidence of D1, D2 and D3 lymphatic collector metastases and frequencies of D1 collector metastases depending on distance from primary tumor);
- proportion of operations which extent is effected by ICG lymphatic mapping (number of patients with change of operation plan due to mapping results divided by the number of patients).

## Methods

### Study population

The study is conducted among male and female adult patients with diagnosed primary colon cancer T1-4aN0-2bM0-1b and clinical indications for elective colonic resection. All patients should sign an informed consent, meet inclusion criteria, and have no exclusion criteria.

### Eligibility criteria

#### Inclusion criteria

- Pathologically confirmed adenocarcinoma of colon (caecum, ascending colon, hepatic flexure, transverse colon, splenic flexure, descending colon, sigmoid);
- TNM – T1-4aN0-2bM0-1b;
- clinical indications to colonic resection;

ECOG – 0–2;

- signed informed consent.

#### Exclusion criteria

- Medical or psychiatric reasons interfering with patient’s decision to participate in the study;
- pregnancy or breastfeeding;
- age less than 18 years;
- medical conditions contraindicating elective surgical procedure;
- acute bowel obstruction, bleeding, perforation, or other tumor complications affecting urgency of surgical procedure;
- peritoneal carcinomatosis;
- invasion of adjacent organs according to preoperative imaging done up to 30 days prior to surgery;
- hypersensitivity to indocyanine green, sodium iodide or iodine;
- hyperthyroidism or autonomic thyroid adenomas;
- kidney failure of any etiology;
- hepatic failure of any etiology;
- poorly tolerated indocyanine injection in the past.

### Quality control

#### Quality control of surgical procedures and ICG lymphatic mapping

To provide routine quality control and ensure possibility of intraoperative data assessment surgical procedures are recorded, and recordings are stored to ensure their availability for revision during data monitoring and in cases of adverse events (Fig. 2). Also, all fresh specimens are photographed after marking of lymph node groups and margins of ICG spread.

#### Morphological assessment

Groups of lymph nodes and margins of indocyanine lymphatic spread are marked on non-fixed specimen by a member of a surgical team taking part in the procedure and determined by surgeon in charge of clinical case. Lymph nodes are marked according to Japanese classification^[15]^ depending on performed extent of colonic resection and lymphadenectomy (Fig. 3).

Specimen assessment is performed according to principles, described by N. West *et al*^[17,18]^, protocol of pathology assessment is described in Appendix S2 http://links.lww.com/ISJP/A8 section.

### Patient treatment

#### Preoperative care

Preoperative investigations and treatment of co-morbidities are conducted according to center’s guidelines and protocols. Preoperative staging is to include, but not to be limited to colonoscopy with biopsy, IV contrast CT chest and abdomen (or abdominal MRI if CT is contraindicated), histological confirmation of cancer and cancer embryonic antigen level. In cases of M+, treatment plan of metastases is to be formulated and documented before enrollment. Results of investigations, fitness for surgery and the fact of meeting or not meeting the eligibility criteria are documented in case report form.

In cases of female patients who are not in menopause (having periods now or in cases when the last period was less than 2 years before enrollment), a pregnancy test is performed, and its results are recorded.

#### Patient enrollment

Patients are enrolled by investigating physician. The study in general and the intervention itself together with information about indocyanine green and associated risk are discussed with the patient, and time and opportunity are provided to allow the patient to familiarize with the informed consent. Should any questions arise, they are answered by investigating physician, and enough time for decision making is provided to the patient. After informed consent is signed, patient is considered as enrolled into the trial.

Patients with signed informed consent, meeting all inclusion criteria and having no exclusion criteria are assigned to elective colonic resection with intraoperative ICG lymphatic mapping. Surgeon in charge decides on extent of colonic resection and planned approach.

Data regarding patients’ demographics, tumor’s TNM, size, and location as well as planned surgery are recorded in case report form.

#### Surgical procedure

Surgical operation is performed under general anesthesia with or without thoracic epidural block. Lymphatic mapping procedure and fluorescence assessment are described in Appendix S1 http://links.lww.com/ISJP/A7 section. Date of surgery, data on operating surgeon and surgeon performing ICG mapping, and technical defects of ICG injection, including spillage, are recorded. Presence and time until pericolic fluorescence, fluorescence along feeding vessels, and aberrant fluorescence (if any is present), influence of ICG spread on resection margins, and intraoperative complications are traced and recorded in case report forms as well.

#### Pathology report

Pathological examination includes macroscopic and microscopic assessments, described in Appendix S2 http://links.lww.com/ISJP/A8 section. During pathological examination each lymph node is assessed both for presence of metastases and for its exact location in relation to primary tumor and margins of indocyanine distribution.

#### Postoperative care

Postoperative care is provided according to local guidelines. Adverse events are registered according to Clavien–Dindo classification. Treatment for complications follows local standards. Data on beginning, duration and treatment for complications is documented in patients’ case report form. Time frame for recording of adverse events is 30 days after surgery. No follow-up is done after this point.

#### Efficacy and safety

Efficacy and safety of surgical procedures are assessed based on pathology reports and postoperative morbidity and mortality. Safety analysis is performed every 25 enrolled patients. Should deviation of morbidity and mortality or frequency of adverse events exceed 30% in intervention group compared to average center statistics for colonic resections, principal investigator and research physician will consider trial discontinuation.

Surgery risks, preferable approach and conversion decisions are surgeon’s responsibility.

In current study adverse event is any unfavorable or unwanted deviation including laboratory tests or condition not necessarily associated with surgery; any change from postoperative state of patient, including exacerbation of co-morbidities. Serious adverse event – any adverse event resulting in death, life-threatening condition, requiring hospital admission or prolongation of in-hospital treatment, permanent disability, or incapacity. Adverse events classification is performed in accordance with CTCAE v. 5.0 terminology.

### Data collection, management and analysis

#### Data quality

Data is entered to electronic CRF (eCRF) by data manager and verified by research physician after enrollment of every 25 patients. Details of surgical procedures are verified by comparing entered data to procedure recordings. All patients’ data is monitored to achieve verification of 100% of data.

#### Collection, storage and sharing of data

Clinical data (demographics, diagnosis, investigations, surgery data, pathology, lab results, etc.) is entered to eCRF basing on primary medical documentation (patient files, outpatient files, investigation reports, laboratory reports, operation notes etc.).

Data in eCRF is entered via google forms and stored under unique identification numbers. Access to data tables is password protected. Only principal investigator and research physician have access to data tables, which are stored for at least 15 years after the end of the study.

After verification data is exported for statistical analysis using SPSS software.

Publications of protocol, first safety report and results in relevant journals are planned. The data will also be shared among specialists via presentations on conferences.

### Sample size calculation and statistical analysis

As the study is aimed to access if ICG mapping can be used to reliably define resection margins in colon cancer surgery, a case of affected lymph nodes only within fluorescence margins was regarded as a true positive case, no fluorescence in pN0 – true negative, pN+ because of metastatic lymph nodes outside fluorescence zone – false negative, pN0 despite presence of fluorescence – false positive.

Sample size of 101 patients was calculated using Buderer method^[19]^ with a confidence level (1 – *α*) of 0.95 as a minimum of cases required to test accuracy of lCG lymphatic mapping for estimated sensitivity of 0.99 and precision (margin of error) of 0.03. This resulted in sensitivity threshold of 0.96. The calculation was performed for minimum acceptable kappa (k0) of 0.6, expected kappa (k1) 0.9. The resultant estimated power (1 – *β*) is 0.96. The average of pN+ cases in our center (42%) was used as prevalence – proportion of outcome (p).

For statistical analysis of primary endpoint, sensitivity will be calculated according to standard formula (number of true positive cases divided by sum of true positive and false negative cases) and its meaning will be compared to the estimated 0.99 ± 0.03. For analytical purposes other measures of accuracy will be calculated as well.

“Intent-to-treat” analysis will be performed, thus once enrolled patients are to be analyzed, irrespective of received treatment. To focus on specificities ICG spread regardless of influence of patients with treatment deviations and those with unsuccessful ICG injection, “per-protocol” analysis will be performed as well.

For secondary endpoints type of analysis will depend on nature of the data. Descriptive statistics will be used to depict feasibility of ICG lymphatic mapping, incidence of adverse events, incidence of lymph node metastases outside conventional resection margins and proportion of operations which extent is affected by ICG lymphatic mapping. Lymph node metastases pattern will be assessed as calculation of frequency of affected nodes related to number of studied nodes in each group. Parametric and non-parametric criteria will be used to compare mean values and contingency tables.

In cases of missing data, should more than 10% of data be missing, the variable will be deleted, because estimates are likely to be biased^[20]^. In other situations multiple imputations will be used.

## Data Availability

Not applicable.

## Control of study conduct

Study conduction is controlled by monitoring timing and quality of CRF completion according to "Efficacy and safety" and "Data quality" parts of the protocol. The protocol of the study and all amendments, as well as informed consent are to be approved by Ethical committee of FSBI "NMRC of Oncology named after N.N. Petrov" of the MoH of the Russian Federation. After amendments are approved, new version of protocol with changes description is spread among trial team.

## Overseeing the study responsibilities

*Principal investigator and research physician*: Design and conduct of the trial, Preparation and approval of protocol and revisions, Preparation of case report forms (CRFs), Control of quality of study conduct, Monitoring of data, Publication of study reports.Data manager: Maintenance of trial IT system and data entry, Data verification.

## Research registration unique identifying number (UIN)

Trial registration number and date of registration: ClinicalTrials.gov NCT05468827, 21.07.2022. Trial registration information is presented in Table 1.
